# Surgical Delay due to Ethylenediaminetetraacetic Acid-Induced Pseudothrombocytopenia

**DOI:** 10.7759/cureus.9273

**Published:** 2020-07-19

**Authors:** Himal Kharel, Nishan B Pokhrel, Samriddha R Pant, Suraj Shrestha, Bishal Agrawal

**Affiliations:** 1 Internal Medicine, Tribhuvan University, Institute of Medicine, Maharajgunj Medical Campus, Kathmandu, NPL; 2 Surgery, Tribhuvan University, Institute of Medicine, Maharajgunj Medical Campus, Kathmandu, NPL

**Keywords:** etda, platelet clumps, psuedothrombocytopenia, thrombocytopenia

## Abstract

Automated hematology analyzer uses the Coulter principle leading to different cell types based on their size. Despite being rapid and convenient, it can result in spurious outcomes like ethylenediaminetetraacetic acid (EDTA)-induced pseudothrombocytopenia. EDTA-induced pseudothrombocytopenia is an immunologically mediated phenomenon resulting from a change in the configuration of glycoprotein (GP) IIb/IIIa by EDTA. The consequence is an exposure of hidden epitope that reacts with certain autoantibodies resulting in spuriously low platelet counts when the blood samples are evaluated by automated blood analyzers. Although it is a rare cause of thrombocytopenia, if not recognized, it can result in unnecessary investigations and treatments. In this case, EDTA-induced psuedothrombocytopenia delayed laparoscopic cholecystectomy planned for symptomatic cholelithiasis in a 58-year-old male. The presence of large platelet clumps on peripheral smear and normal manual platelet counts confirmed the diagnosis. Pseudothrombocytopenia should be suspected when there is no correlation between clinical and laboratory findings in a patient with a low platelet count. Reperforming counts with other anticoagulants and if necessary, manual count in the peripheral blood smear is suggested.

## Introduction

Thrombocytopenia is defined as a platelet count lower than 150,000/mm^3^ [1]. Pseudothrombocytopenia (PTP) is defined by falsely low platelet counts on automated analyzers and is caused by in vitro phenomena including large platelet aggregates in blood samples. Platelet aggregates on account of their large size are frequently not included in the platelet window of autoanalyzers, leading to misleadingly low platelet count [2]. Dipotassium EDTA (K2-EDTA) was used in the form of spray-painted vacutainer tubes in our institution. EDTA-induced PTP is a result of a configurational change in glycoprotein (GP) IIb/IIIa by EDTA and the presence of certain specific autoantibodies.

Here, we are reporting a case of a middle-aged gentleman diagnosed with symptomatic cholelithiasis whose laparoscopic cholecystectomy was delayed due to EDTA-induced PTP. The operation was successful, and he was discharged after an uneventful postoperative period.

## Case presentation

A 58-year-old male with no personal or family history of bleeding had intermittent right upper quadrant pain for two years which occurred with heavy fatty meals. He was not exposed to heparin in the past. At the time of presentation, the abdomen was soft, and non-tender with audible bowel sounds. Liver and spleen were not enlarged. Ultrasonography of the abdomen showed multiple stones in the gallbladder, with the largest measuring nine millimeters. There was no wall thickening and pericholecystic collection.

Symptomatic cholelithiasis was diagnosed, and laparoscopic cholecystectomy was planned. Routine preoperative investigations showed normal findings except for a low platelet count of 27,000/mm^3^. The surgery was postponed due to low platelet count and the patient was followed up on an outpatient clinic.

**Figure 1:**
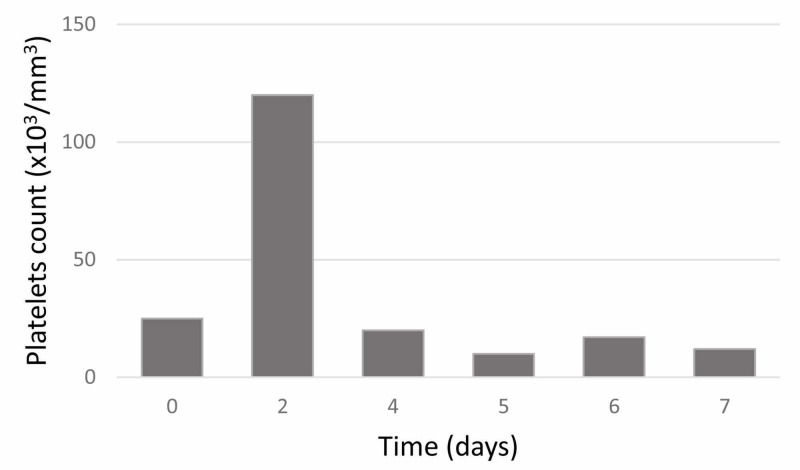
Changes in platelet counts during outpatient visits prior to the surgery

Platelet counts were repeatedly checked for a week (Figure 1).

The platelet count varied from 10,000 to 127,000/mm^3^. As the patient had no bleeding manifestations like petechiae, purpura, epistaxis, gum bleeds, or melena, and due to highly fluctuating platelet counts, PTP was suspected. Hence, manual platelet count was performed in the peripheral blood smear which showed the presence of platelet clumps. It showed adequate platelets with an estimated count of 197,000/mm^3^. Therefore, the patient was cleared for laparoscopic cholecystectomy surgery. The postoperative period was uneventful. He was discharged on the fourth postoperative day when he was in good physical condition and tolerated his diet. He had no issues during follow-up one month after surgery.

## Discussion

**Figure 2:**
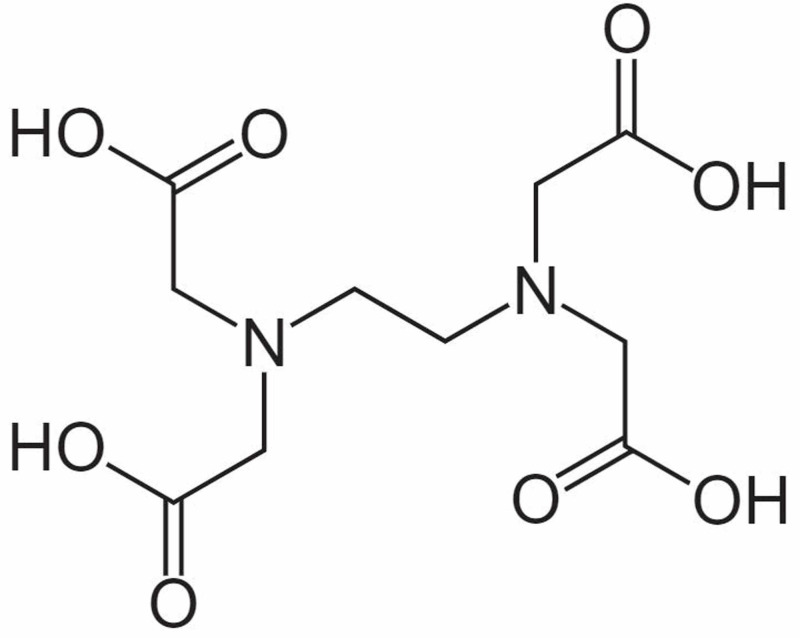
Chemical structure of EDTA EDTA, ethylenediaminetetraacetic acid

EDTA, chemically known as 1,2-bis[bis(carboxymethyl)amino]ethane, is one of the best anticoagulants for in vitro testing of hematological parameters of blood because it allows the best preservation of cellular components and morphology of blood cells [3]. It has an anion with long tentacle-like groups that can wrap around metal ion (Figure 2).

The complex ions that form between polydentate ligands and cations are known as chelated complexes whose name came from the Greek word ‘chela' meaning 'a crab's claw'. This property of EDTA is used in the medical field for the prevention of clotting of blood by chelating the calcium ions.

Similarly, citrate (3.2%) is also used as an anticoagulant especially for coagulation assays but not for hematological studies because it alters the cellular morphology. But, as it does not alter GP IIb/IIIa, it can be used in cases of EDTA-induced thrombocytopenia to obtain almost accurate platelet counts. Lippi and Plebani have proposed a criterion for diagnosing EDTA-induced thrombocytopenia [4].

**Figure 3:**
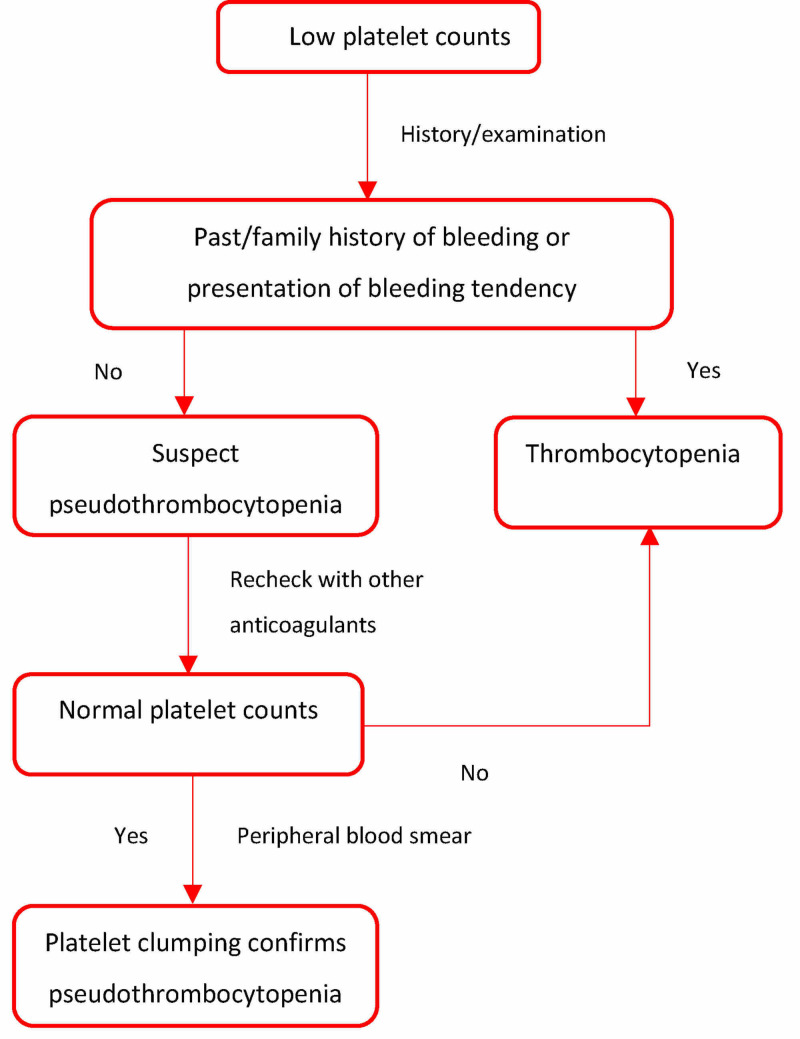
Approach to low platelet counts

The following algorithm might help differentiate thrombocytopenia from PTP when we encounter low platelet counts (Figure 3).

The causes of thrombocytopenia are varied, some of which are immune thrombocytopenic purpura (ITP), bone marrow hypoplasia, leukemia, chronic liver disease, drug-induced thrombocytopenia, and disseminated intravascular coagulation (DIC) [5]. Ruling these causes out is of utmost priority, especially if the patient is symptomatic. Detailed history taking, including drug history and family history, and physical examination, to look for lymphadenopathy and hepatosplenomegaly, must be performed. Complete blood counts, peripheral blood smears, and bone marrow biopsy need to be done to determine whether the abnormality is within one cell line or generalized among different blood cell lineages. These also help rule out bone marrow hypoplasia and leukemia [5]. Deranged prothrombin time, fibrinogen, and D-dimers would point towards DIC. Liver function test can be performed to rule out hepatic causes of thrombocytopenia. Serological tests for viruses and blood cultures can be sent to rule out infections. ITP could be a diagnosis of exclusion where only platelet as a cell line has been affected. As the presented case was asymptomatic, these tests (with the exception of routine blood workup mentioned before) were not performed [5].

PTP is a relatively rare phenomenon that can result from clotting of the blood sample in a test tube, platelet satellitism, presence of large platelets, and EDTA-induced thrombocytopenia. PTP is not only seen in healthy individuals, but also reported in association with autoimmune, cardiovascular and liver parenchyma diseases, malignancy, sepsis, viral infection, and some medications [6-8]. The pathophysiology of EDTA-dependent PTP has been described below.

Platelet cohesion depends on the interaction between GP IIb/IIIa and fibrinogen. About 0.1% to 2% of hospitalized patients have IgG or IgM antibodies against a hidden epitope in GP IIb/IIIa which is typically inconsequential in vivo [9]. These antibodies can be associated with occult malignancies, antinuclear antibodies, antibiotics like levofloxacin and ceftriaxone, and procedures like transcatheter arterial embolization of hepatocellular carcinoma [6,7,10]. In vitro EDTA exposure changes the configuration in GpIIb/IIIa and exposes the epitopes where the IgG or IgM antibodies bind [1]. The platelets aggregate in clumps and are counted as leukocytes by the blood analyzers, which distinguish between cells based on their size, leading to falsely low values of platelets and spuriously high leukocyte counts. This phenomenon is never seen in patients with Glanzmann's thrombasthenia who inherently lack GP IIb/IIIa [3]. It can be prevented by using citrated or heparinized blood samples, heating the samples to 37°C, adding aminoglycosides to the blood samples, or counting platelets manually as was done in this case [11]. The exact mechanism of aminoglycosides in preventing EDTA-associated thrombocytopenia is unknown but it has been shown to prevent and cause dissociation of aggregated platelet clumps [11].

## Conclusions

PTP should be considered after excluding other common causes of thrombocytopenia, when low platelet counts do not correlate clinically. Reperforming counts with other anticoagulants and if necessary, manual counts in a peripheral blood smear are suggested. Early detection prevents needless investigations and treatments, and their risks.

## References

[1] Hillman RS, Ault KA, Leporrier M, Rinder HM (2010). Thrombocytopenia. Hematology in Clinical Practice (LANGE Clinical Medicine).

[2] Kamath V, Sarda P, Chacko MP, Sitaram U (2013). Pseudothrombocytopenia observed with ethylene diamine tetraacetate and citrate anticoagulants, resolved using 37°C incubation and kanamycin. Indian J Pathol Microbiol.

[3] Banfi G, Salvagno GL, Lippi G (2007). The role of ethylenediamine tetraacetic acid (EDTA) as in vitro anticoagulant for diagnostic purposes. Clin Chem Lab Med.

[4] Lippi G, Plebani M (2012). EDTA-dependent pseudothrombocytopenia: further insights and recommendations for prevention of a clinically threatening artifact. Clin Chem Lab Med.

[5] Izak M, Bussel JB (2014). Management of thrombocytopenia. F1000Prime Rep.

[6] Kinoshita Y, Yamane T, Kamimoto A, Oku H, Iwata Y, Kobayashi T, Hino M (2004). A case of pseudothrombocytopenia during antibiotic administration. (Article in Japanese). Rinsho Byori.

[7] Abe H, Shimizu T, Cho H, Kubota Y, Umeda T, Kurumi Y, Tani T (2010). Occult breast cancer with EDTA-dependent pseudothrombocytopenia-a case report. Gan To Kagaku Ryoho.

[8] Kim HJ, Moh IH, Yoo H (2012). Ethylenediaminetetraacetic acid-dependent pseudothrombocytopenia associated with neuroendocrine carcinoma: a case report. Oncol Lett.

[9] Isik A, Balcik OS, Akdeniz D, Cipil H, Uysal S, Kosar A (2012). Relationship between some clinical situations, autoantibodies, and pseudothrombocytopenia. Clin Appl Thromb Hemost.

[10] Yoshikawa T, Nakanishi K, Maruta T (2006). Anticoagulant-induced pseudothrombocytopenia occurring after transcatheter arterial embolization for hepatocellular carcinoma. Jpn J Clin Oncol.

[11] Sakurai S, Shiojima I, Tanigawa T, Nakahara K (1997). Aminoglycosides prevent and dissociate the aggregation of platelets in patients with EDTA‐dependent pseudothrombocytopenia. Br J Haematol.

